# Outcome after 52 Salto Ankle Prostheses Implanted by a Single Surgeon

**DOI:** 10.1155/2018/2735634

**Published:** 2018-08-01

**Authors:** Frank W. M. Faber, Monique J. L. Mastboom, Sabine T. van Vliet-Koppert, Ilse C. E. Bouman, Paulien M. van Kampen

**Affiliations:** ^1^Department of Orthopaedic Surgery, Bergman Clinics, Rijswijk, Netherlands; ^2^Department of Orthopaedic Surgery, LUMC, Leiden, Netherlands; ^3^Department of Orthopaedic Surgery, HAGA Hospital, The Hague, Netherlands

## Abstract

While ankle arthrodesis was traditionally the gold standard method of treatment for disabling end-stage ankle arthritis, total ankle replacement (TAR) has been an acceptable alternative. The satisfaction rate of patients with TAR however differs. The purpose of our study is to investigate whether implant survival and results with special emphasis on the satisfaction rate of patients treated with a TAR implanted by a single surgeon were comparable to the literature. This was a retrospective cohort study in a teaching hospital. Data was collected from 52 patients who received a total ankle replacement (TAR) between 05/2002 and 06/2014. The mean follow-up time was 4.2 years (95% CI 3.3 – 5.0). Results showed a high satisfaction rate of 94% and 94% survival of the TAR after 5 years. We conclude that TAR with the Salto prosthesis is, in our hands, a reliable solution for end-stage ankle arthritis, with results comparable to the literature.

## 1. Introduction

While ankle arthrodesis was traditionally the gold standard method of treatment for disabling end-stage ankle arthritis, total ankle replacement (TAR) has been an acceptable alternative [1–4]. Drawbacks of ankle fusion are nonunion, quite long postoperative immobilization, and in the long-term arthritis in the adjacent joints [5, 6]. Disadvantages of TAR are the technical difficulty of the procedure, causing a considerable learning curve [7–9] and uncertain long-term survival of the implant [3, 10, 11]. The satisfaction rate of patients with TAR also differs. Conflicting reports exist about the cause for this difference in satisfaction. Spirt et al. [12] and Henricson et al. [13] found a worse result in younger patients, focusing on implant survival, complication rates, and revision rates. Tenenbaum et al. [14] and Dematracopoulous et al. [15] reported equal results in different age groups, considering clinical outcomes, gait improvement, and patient reported outcome. Schenk et al. [16] reported 6.9% unsatisfied patients, mostly because of conversion of the prosthesis to an arthrodesis. Other factors influencing implant survival, and with that patient satisfaction, are preoperative alignment, body weight, and proper operative technique. Furthermore, management of the expectations of the patients and explicit information in the preoperative phase is of influence [17]. The purpose of our study is to investigate whether implant survival and results with special emphasis on the satisfaction rate of our patients treated with a TAR were comparable to the literature.

## 2. Patients and Methods

### 2.1. Study Design and Setting

This was a retrospective cohort study. Data was collected from patients who received a total ankle replacement (TAR) between 05/2002 and 06/2014 in the HAGA Hospital, the Hague, in the Netherlands. All surgeries were performed by the same Orthopedic Surgeon specialized in foot and ankle surgery (FF). The study was enlisted by the Medical Ethical Committee Southwest Holland (15-103) and was declared not to subdue to Medical Research Involving Human Subjects Act. The board of directors of the hospital approved the study.

### 2.2. Participants

Patients who received a TAR in the previous mentioned time window were included in the study. Patients with the following criteria were included: symptomatic ankle osteoarthritis (Kellgren-lawrence score of 3+ or 4 despite conservative treatment), age≥ 65 years (except rheumatoid patients), BMI ≤ 30, American Society of Anesthesiologists (ASA) stage 1, 2, or 3, adequate bone-quality, ≤10° varus or valgus deformity, and no (extreme) sport wish. Exclusion criteria for a TAR were neurological disorders, bad peripheral circulation, osteonecrosis of talus or tibia, and infection present. The operation was performed under spinal or general anesthesia. Half an hour prior to surgery and 24 hours postoperatively intravenous antibiotics were administered. Standard anterior approach was used and an uncemented SALTO® prosthesis (Tornier SA, Saint Ismier, France) was placed (a third-generation ankle prosthesis, three components, and mobile bearing). Postoperative treatment consisted of a lower leg cast: first 2 weeks non-weight-bearing followed by 2 weeks full-weight-bearing. After cast removal, an intensive physical therapy program was followed for at least 6 weeks. The questionnaires were distributed with a minimum of 1 year follow-up and the postoperative X-rays were made the first day after surgery and 4 weeks postoperatively. The follow-up X-rays were performed with AP and lateral weight-bearing X-rays on a 2-yearly routine base.

### 2.3. Variables

Patients' satisfaction was measured by asking one question: “would you choose the TAR again in a similar situation?”

Revision of the TAR was defined as any secondary operation in which a prosthetic component was exchanged and/or conversion to an arthrodesis had to be performed, for any reason.

Functional outcome was measured by the Foot Function Index (FFI) [18]. We used the validated Dutch Version using verbal rating scales provided by Kuyvenhoven et al. [19]. The second outcome measure was the American Orthopaedic Foot and Ankle Society (AOFAS) questionnaire [20].

### 2.4. Measurement

**Figure 1:**
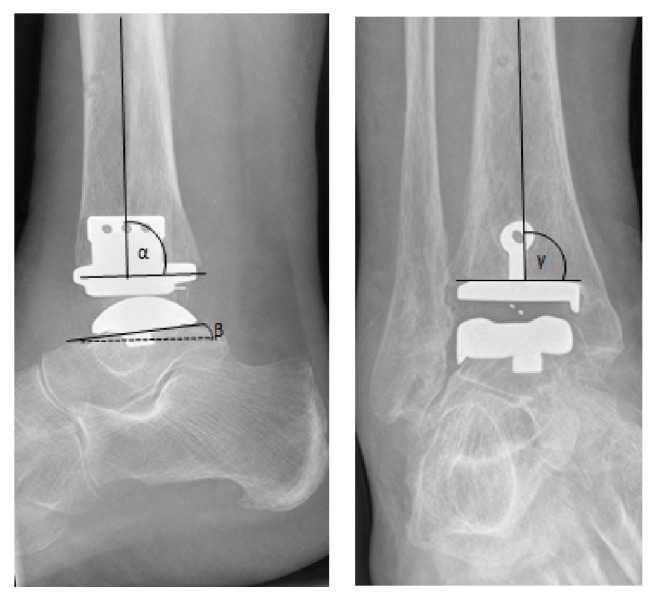
Radiological angles. Perfect tibial position is defined as *γ* angle 90 degrees and in the sagittal plane position of the tibial component with an inclination till 7 degrees (*α* = +7; according to the manufacturer of the Salto prosthesis). Perfect talar position is placement of this component parallel to the sole of the foot (*β* angle 0 degrees). An angle-difference over time of 5 degrees or more is considered significant and is classified as migration. Malposition is defined as an angulation of a component of more than 10 degrees from the perfect position as defined by Schimmel [7].

Radiological outcome for the position of the prosthesis components is measured in degrees on first weight-bearing postoperative X-ray compared to last postoperative X-ray (Figure 1). Measurements, calculated as angular deviation according to the perfect position, are performed as described by Valderrabano et al. [21].

Radiographic analysis were performed by one independent senior orthopaedic surgeon (SvVK).

### 2.5. Statistical Methods

Continuous baseline characteristics were presented as mean with 95% confidence interval between square brackets. Nominal data was presented in counts and percentages. Statistical analyses were conducted in SPSS version 17 (IBM Co., Armonk, NY, USA). For all statistical analyses, a two-tailed p-value less than 0.05 was considered to be statistically significant.

Patient satisfaction was presented in percentages. A Kaplan-Meier curve was presented for survival analysis with revision for any reason as endpoint. All functional outcomes were presented as mean with 95% confidence interval (CI) and the radiological outcomes as median and IQR.

## 3. Results

### 3.1. Participants

A total 52 patients were included in the study. The mean follow-up time was 4.2 years (95% CI 3.3 – 5.0, range 0.1 – 13.7).

All baseline characteristics are presented in Table 1.

### 3.2. Outcome

In total 48 of the 51 patients would choose the TAR again in a similar situation (94% satisfaction rate). One answer was missing, because we were unable to contact this patient. Two patients were dissatisfied with the TAR; one patient required a conversion to an arthrodesis for septic loosening and the other dissatisfied patient had persistent pain, but had good function and perfect radiological images. This TAR was converted to an ankle arthrodesis for suspicion of low grade infection, which was not confirmed. Pain persisted even after an uneventful solid ankle fusion with allograft.

Three of all patients had an ankle arthrodesis on the contralateral side. These patients all preferred their TAR over their ankle arthrodesis.

**Figure 2:**
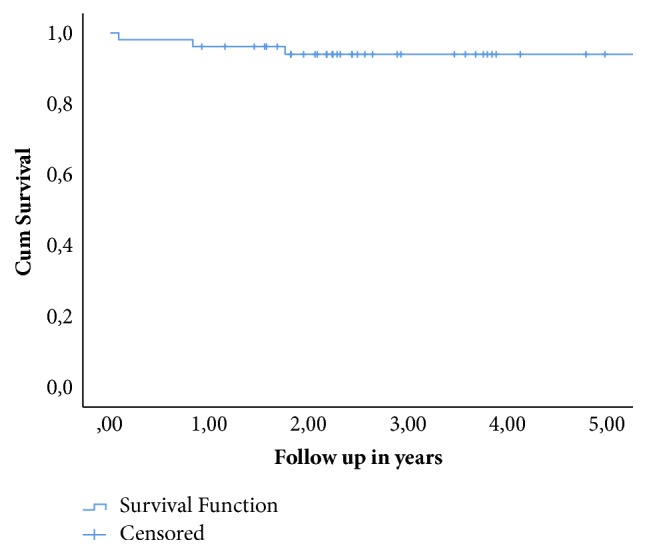
Survival of the TAR in years. At 2,5 years 29 were at risk and at 5 years 15 were at risk. The overall survival time is 11 with a 95% confidence interval of 10-13.

In Figure 2 the Kaplan-Meier curve is presented. The survival of the TAR after 5 years is 94% with 15 patients at risk.

The functional and radiological results are presented in Table 2. The mean total AOFAS score was 85 out of a maximum of 100 points. The mean total FFI was 36 out of a maximum of 115 (with 23 as lowest and best score).

Not for all the 52 patients clinical and radiological data could be collected. The patients that were converted to an arthrodesis were excluded. In addition, we were unable to reach one patient (like mentioned above) and one patient was not willing to come to the hospital and only answered the satisfaction question on the phone. This explains the different numbers in the first column.

### 3.3. Complications

Five talar component malpositions were noted at postoperative weight-bearing X-rays. Four malpositions of the tibial part were discovered, of which one was treated with a reoperation within two weeks: repositioning of the tibial component leading to a satisfactory result afterwards. Preoperatively, 2 medial malleolus fractures and 1 lateral malleolus fracture occurred. All fractures were fixed during the ankle replacement procedure.

Three patients suffered from prolonged wound healing: more than 4 weeks postoperatively wound leakage was present: these all resolved with prolonged immobilization and antibiotics.

During the follow-up period in 4 cases, conversion to an arthrodesis was necessary. Two patients needed conversion because of aseptic loosening caused by multiple cysts; one patient suffered from severe wound problems and an acute deep infection. One conversion was performed because of persistent pain and suspicion of low grade infection, which was not confirmed during reoperation.

## 4. Discussion

TAR is known as a technically demanding procedure and a considerable learning curve is described by several authors [7–9]. Yet, equally functional results are described by Reuver et al. [22] in low volume centres: the AOFAS score of these authors had an average of 75 (SD ± 15). Our study, single surgeon and low volume, shows an even higher average AOFAS score of 85 (SD ± 17). This difference could be explained because the senior author of this series (FF) already had considerable experience in TAR with another type ankle prosthesis (STAR) before he started implanting the Salto prosthesis. The functional outcome of this study is comparable to other series that also described the FFI and/or the AOFAS score as an outcome parameter. The FFI found by Kerkhoff et al. [23] and Schimmel et al. [7] were 33 and 32, respectively. The AOFAS score varied from 73 to 85 [16, 24–27]. Bonnin, who is actually one of the inventers of the Salto prosthesis, described an AOFAS score of 79 points, with a follow-up of 7–11 years [28]. So, in our hands, the functional results are certainly not inferior.

In our study 48 of the 51 patients (94%) would have the TAR performed again in a similar situation, of whom some are even revised and converted to an arthrodesis. Yet, 2 of the 2 dissatisfied patients had a revision. So implant survival and satisfaction are definitely related. Implant survival can be improved by proper technique, for instance, component placement in proper alignment [3, 29]. Our survival rate is 94% after 5 years with 15 patients at risk. This is better than the results described by Henricsson et al. [13], that is, 78% survival at 5 years. One study found a 94% implant survival at 5 years, even 87% survival at 10 years, which dropped quite steep to 64% after 15 years [30]. One of our exclusion criteria for a TAR is age: we excluded patients younger than 65 years, in whom we prefer an arthrodesis, except for rheumatoid patients. These patients often have other joint impairments (hindfoot, midfoot), which probably benefit when the ankle joint is kept mobile instead of fused. Gait analysis showed a more normal gait in TAR patients than in patients who had an arthrodesis [31]. Although the age selection criterion seems justifiable in terms of long-term implant survival, reports in the literature are conflicting. Kofoed and Lundberg-Jensen [32] and Skyttä et al. [33] described no influence of age; Tenenbaum et al. [14] reported equal functional improvement in patients over 70 years patients and aged 50-60 years. In contrast with this, Henricson et al. [13] and Raikin et al. [34] all reported worse results in younger patients. Because of our high patient satisfaction and the good survival rate compared with the literature, we consider performing TARs in younger patients. We realize we have to inform this younger group about the risk of a conversion to an arthrodesis later in life.

The strong points of this study were the independent investigators who performed the patient investigations at follow-up, the high follow-up percentage, and the use of one and the same prosthesis, with an unchanged design by the same surgeon. A weak point is its retrospective design, so no preoperative clinical scores were available.

## 5. Conclusion

We conclude that TAR with the Salto prosthesis is, in our hands, a reliable solution for end-stage ankle arthritis, with results comparable to the literature.

## Figures and Tables

**Table 1:** Baseline characteristics.

**Age, mean**	Years, range	70 [49 – 86]

**Gender**	Male	26 [50%]

	Female	26 [50%]

**Side**	Left	19 [64%]

	Right	33 [37%]

**ASA**	1	21 [40%]

	2	26 [50%]

	3	2 [4%]

	Missing	3 [6%]

**Smoking**	No	44 [85%]

	Yes	7 [14%]

	Missing	1 [2%]

**Indication**	Post-traumatic	29 [56%]

	Primary Osteoarthritis	18 [35%]

	Rheumatoid Arthritis	4 [8%]

	Rheumatoid Arthritis and Post-traumatic	1 [2%]

**Table 2:** 

		N			Range
**FFI**	Activity	45	Mean [95% CI]	18 [16 -20]	[9-43]

	Pain	45	Mean [95% CI]	16 [14 – 18]	[9-39]

	Restriction	45	Mean [95% CI]	8 [7 -9]	[5-16]

	Total	45	Mean [95% CI]	42 [37 – 47]	[25-94]

**AOFAS**	Alignment	45	Mean [95% CI]	9 [8 – 10]	[0-10]

	Function	45	Mean [95% CI]	41 [38 – 45]	[5-50]

	Pain	45	Mean [95% CI]	34 [32 – 37]	[20-40]

	Total	45	Mean [95% CI]	85 [80 – 90]	[26-100]

**Radiological outcome**	First alpha	49	Median [IQR]	6 [4]	[ -3 - 12]

	Last alpha	47	Median [IQR]	5 [4]	[-3-14]

	First beta	49	Median [IQR]	-4 [8]	[-15-9]

	Last beta	47	Median [IQR]	-4 [9]	[-15-8]

	First gamma	49	Median [IQR]	0 [2]	[-4-5]

	Last gamma	47	Median [IQR]	0 [4]	[-5-7]

## Data Availability

The data used to support the findings of this study are available from the corresponding author upon request.
